# Inhibition of Human Transthyretin Aggregation by Non-Steroidal Anti-Inflammatory Compounds: A Structural and Thermodynamic Analysis

**DOI:** 10.3390/ijms14035284

**Published:** 2013-03-06

**Authors:** Ricardo O. Sant’Anna, Carolina A. Braga, Igor Polikarpov, Salvador Ventura, Luis Mauricio T. R. Lima, Debora Foguel

**Affiliations:** 1 Institute of Medical Biochemistry, Structural Biology Program, Federal University of Rio de Janeiro, Rio de Janeiro, RJ 21941-590, Brazil; E-Mails: santanna@bioqmed.ufrj.br (R.O.S.A.); carolina@xerem.ufrj.br (C.A.B.); 2 Polo de Xerém, Federal University of Rio de Janeiro, Rio de Janeiro, RJ 25245-390, Brazil; 3 Institute of Physics of São Carlos, University of São Paulo, Av. Trabalhador Saocarlense, 400, São Carlos, SP 13566-590, Brazil; E-Mail: ipolikarpov@ifsc.usp.br; 4 Institute of Biotechnology and Biomedicine and Department of Biochemistry and Molecular Biology, University of Barcelona, Bellaterra (Barcelona) 08193, Spain; E-Mail: salvador.ventura@uab.es; 5 Faculty of Pharmacy, Federal University of Rio de Janeiro, Av. Carlos Chagas Filho 373, CCS, Bss34, Ilha do Fundão, Rio de Janeiro, RJ 21941-902, Brazil; 6 Laboratory for Biotechnology (LaBio-DIPRO), Brazilian National Institute of Metrology, Quality and Technology—INMETRO, Av. N. Sa. das Graças, 50-Xerém, Duque de Caxias, RJ 25250-020, Brazil

**Keywords:** transthyretin, protein aggregation, high hydrostatic pressure, crystallography, inhibitors

## Abstract

Transthyretin (TTR) is a homotetrameric protein that circulates in plasma and cerebral spinal fluid (CSF) whose aggregation into amyloid fibrils has been associated with at least two different amyloid diseases: senile systemic amyloidosis (SSA) and familial amyloid polyneuropathy (FAP). In SSA aggregates are composed of WT-TTR, while in FAP more than 100 already-described variants have been found in deposits. Until now, TTR-related diseases have been untreatable, although a new drug called Tafamidis has been approved only in Europe to specifically treat V30M patients. Thus, new strategies are still necessary to treat FAP caused by other variants of TTR. TTR has two channels in the dimer interface that bind to the hormone thyroxin and that have been used to accommodate anti-amyloidogenic compounds. These compounds stabilize the tetramers, rendering TTR less amyloidogenic. Here, we investigated the effects of three non-steroidal anti-inflammatory compounds—sulindac (SUL), indomethacin (IND) and lumiracoxib (LUM)—as tetramer stabilizers and aggregation inhibitors. WT-TTR and the very aggressive TTR variant L55P were used as models. These compounds were able to stabilize TTR against high hydrostatic pressure (HHP), increasing the ΔG_f_ by several kcal. They were also effective in inhibiting WT-TTR and L55P acid- or HHP-induced aggregation; in particular, LUM and IND were very effective, inhibiting almost 100% of the aggregation of both proteins under certain conditions. The species formed when aggregation was performed in the presence of these compounds were much less toxic to cells in culture. The crystal structures of WT-TTR bound to the three compounds were solved at high resolution, allowing the identification of the relevant protein:drug interactions. We discuss here the ligand-binding features of LUM, IND and SUL to TTR, emphasizing the critical interactions that render the protein more stable and less amyloidogenic.

Abbreviations

TTR: Transthyretin
FAP: familial amyloid polyneuropathy
SSA: senile systemic amyloidosis
CSF: cerebrospinal fluid
HBS: hormone binding site
CR: Congo red
HHP: high hydrostatic pressure
SEC: size exclusion chromatography
CNS: central nervous system
LUM: lumiracoxib
IND: indomethacin
SUL: sulindac
ANS: 8-anilino-1-naphthalenesulfonic acid

## 1. Introduction

Transthyretin (TTR) is a homotetrameric, β-sheet–rich protein of 56 kDa produced in the liver or in cerebral spinal fluid (CSF). TTR has two channels at the dimer–dimer interface, where the hormone thyroxine and other ligands can bind. These channels are also called hormone binding sites (HBS), although only a low percentage of the TTR that circulates in the plasma or in CSF has its channels occupied by thyroxine [1–4]. Therefore, HBS have been used as targets for the development of drugs that bind and stabilize TTR against dissociation and aggregation [5–13].

TTR is associated with two amyloidoses: senile systemic amyloidosis (SSA) and familial amyloid polyneuropathy (FAP) [14,15]. SSA is caused by the massive deposition of aggregates composed of wild-type TTR (WT-TTR) mainly in the heart [16,17], while FAP is caused by the deposition of aggregates at the peripheral nerves and tissues that are composed of more than 100 autosomal TTR variants [18,19]. Among the identified mutations, the L55P substitution is one of the most aggressive variants and is associated with early onset of the disease with cardiac and neurologic pathologies [20–22].

Thus far, the mechanism that leads to TTR amyloid formation presupposes tetramer dissociation and monomer partial unfolding [23–26], the latter constituting the building blocks for fibril formation. In this context, studies have shown that disease-linked mutations destabilize the TTR tetramer, enhancing the fibril-formation propensity [22,27,28]. *In vitro* studies have used acidic pH to trigger TTR tetramer dissociation and amyloid formation, which led to the hypothesis that, *in vivo*, TTR aggregation would likely take place at the lysosomes [23,24,29,30]. Our group has shown that TTR amyloidogenesis can also be triggered by high-hydrostatic pressure (HHP) treatment resulting in thioflavin-T- and Congo red (CR)-positive aggregates [27,31,32]. SEC experiments demonstrated that the species that undergoes aggregation after HHP release is an altered tetramer called T_4_*. This tetramer presents a decreased thermodynamic stability when compared to the native, non-pressurized tetramer. Together, these data suggest that TTR amyloid formation can use a partially unfolded monomer or T_4_* as a building block, either by itself or with the monomer released because T_4_* stability is compromised [31].

Until recently, the only clinical approach to stem the progression of FAP symptoms was liver transplantation [15,33,34], because this is the organ that produces the majority of TTR that circulates in the blood. Transplantation replaces the organ producing the variant allele with one producing the more stable and less amyloidogenic WT-TTR protein. Transplantation is a very invasive method and cannot be used to treat the rare cases of TTR variants that deposit in the CNS [35–37]. Therefore, there is a need for alternative, noninvasive and broad therapies to tackle TTR-related disorders. Of special impact is Tafamidis, a first-in-class compound designed by Dr. Kelly’s group that has been recently approved only in Europe for the treatment of FAP, in particular for individuals with the V30M mutation [38,39]. In this context, the description of new compounds, in particular non-steroidal anti-inflammatory drugs (NSAI), NSAI analogues and even natural products, all of which are able to bind to HBS and stabilize the tetramers, are proving to be very promising [5–13].

**Figure 1:**
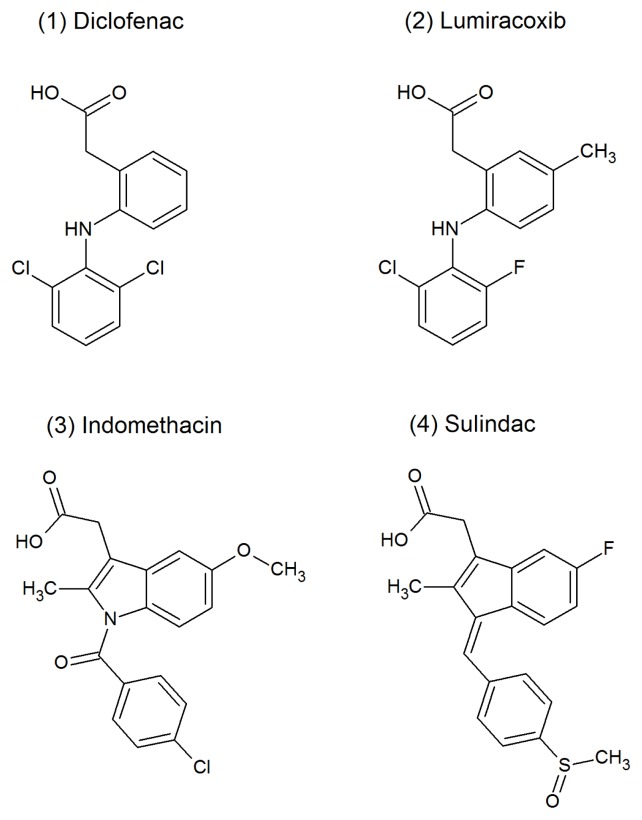
Chemical structures of non-steroidal anti-inflammatory compounds investigated here as possible inhibitors of transthyretin (TTR) aggregation. The structure of diclofenac is also shown for comparison.

Here, we explored the anti-amyloidogenic properties of a group of NSAI, specifically lumiracoxib (LUM), sulindac (SUL) and indomethacin (IND) (Figure 1), whose mechanisms of action have not yet been extensively characterized. Indeed, a preliminary characterization of the effects of IND and SUL but not LUM on WT-TTR aggregation has been reported elsewhere [40,41]. Here, we revisited the inhibitory aggregation properties of these compounds and now report the crystallographic structures of WT-TTR bound to each as well as their mode of binding. Moreover, in addition to using the classical pH-induced aggregation TTR protocol (pH 4.4) [23], we also investigated the effects of these three compounds on the HHP-induced aggregation of TTR, which seems to use T_4_* as a building block. LUM, which is structurally similar to diclofenac, is one of the most effective TTR stabilizers thus far characterized [7,42] and was shown to be the most potent compound, massively inhibiting the pH- or HHP-induced aggregation of L55P and WT-TTR in a 1:2 protein:drug ratio. IND was also a very effective inhibitor, but SUL, even in a high protein:drug molar ratio, was not effective in inhibiting TTR aggregation, particularly L55P aggregation. Interestingly, when aggregation was performed in the presence of these compounds, the species formed were much less toxic to N2a cells than the aggregates generated in their absence. From HHP studies, the thermodynamic parameters for the association-folding of TTR in the presence of SUL and IND (but not for LUM due to methodological limitations) were also calculated, and the results showed that they increased in the ΔG_f_ for WT-TTR and L55P. The data reported here allowed us to correlate tetramer stabilization and aggregation inhibition with structural features that are important for the identification of novel lead compounds for the prevention of TTR-related amyloidoses.

## 2. Results and Discussion

### 2.1. Ligand Binding Enhances Tetramer Stability

Initially, we measured the affinity of LUM, IND and SUL for WT-TTR and L55P using ANS displacement from the HBS [43]. As shown in Figure S1 and Table S1, all compounds displayed apparent dissociation constants in the range between 1 and 7 μM, and LUM had the highest affinities for WT-TTR and L55P (1.4 and 1.7 μM, respectively). Here, we are referring to the affinities as apparent, because TTR has two HBS in each tetramer that ligands generally bind with negative cooperativity [9,38,44,45]. Our experiments did not allow us to discriminate ligand binding between them. The affinity constants of diclofenac, a well-known TTR stabilizer and aggregation inhibitor, for WT-TTR were k_d1_ 0.060 μM and k_d2_ 1.2 μM, which were in the same range as LUM. Tafamidis, the only drug already approved in Europe to treat FAP, exhibited higher affinities for WT-TTR (0.003 μM and 0.154 μM) [7,38,42].

**Figure 2:**
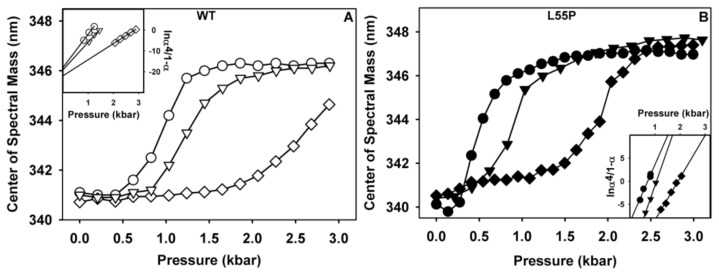
Evaluating the thermodynamic stability of WT-TTR and L55P against HHP in the absence or presence of SUL and IND. WT (panel **A**) or L55P (panel **B**) were incubated at 1 μM in the absence (circles) or presence of 10 μM SUL (triangles) or IND (diamonds) at pH 7.5, 1 °C. Samples were subjected to a stepwise increase in pressure for 10 min at each pressure value before collecting tryptophan emission spectra (excitation = 280 nm and emission = 300 to 400 nm). The insets show the linear regressions from which the thermodynamic parameters were calculated according to Equations 5 and 6 in Material and Methods.

Next, we investigated the effects of these three compounds on tetramer stability. For this purpose, HHP is a suitable variable, because changes in free energy depend exclusively on the volume change (Δ*V*) [31,32,46,47]. Moreover, HHP allowed us to compare the stability among WT-TTR and its variants what is important since stability correlates with aggregation propensity [27]. TTR (1 μM of tetramers, both WT and L55P) was incubated in the absence or presence of IND or SUL (10 μM of each compound) at pH 7.5 and 1 °C and was subjected to increasing pressures, while tryptophan fluorescence emission was measured for each pressure value to evaluate the extent of TTR unfolding. Tryptophan emission is very sensitive to the polarity of the environment where this residue is located. When buried inside the protein interior, such as in the folded state, tryptophan maximum emission is blue shifted. Upon protein unfolding and exposition to the aqueous environment, tryptophan emission shifts to the red (higher wavelengths, thus low energy) loosing energy due to solvent relaxation [48] Thus, the shift of the maximum emission of tryptophan fluorescence to higher wavelengths has been used with confidence to determine the protein folding state [49]. The correlation between TTR tryptophan fluorescence and the degree of denaturation has been previously established [31,50]. Unfortunately, this experiment could not be performed with LUM due to the high fluorescence emission displayed by this compound in the same range as tryptophan emission (not shown). As shown in Figure 2, the dissociation–denaturation of WT-TTR (panel A) and L55P (panel B) induced by HHP in the absence of any addition (circles) was followed by a shift of the tryptophan fluorescence emission to the red by ~5–7 nm (Table 1, ΔCM = delta center of mass). In the presence of SUL (triangles) or IND (diamonds), there was a substantial stabilization of both proteins, which can be seen by the displacement of the denaturation curves to higher pressure values. However, in all cases at elevated pressures, the same extent of denaturation was achieved, with the exception of WT-TTR in the presence of IND (Figure 2A, hollowed diamonds). The changes in the p_1/2_ (pressure value that furnishes 50% change in the center of mass of tryptophan emission) are reported in Table 1, where it is possible to observe the great stability conferred by these compounds to TTR; IND is a greater stabilizer for both proteins (Table 1).

From the data presented in Figure 2, the thermodynamic parameters for folding (Δ*G_f_* and Δ*V_f_*) for WT-TTR and L55P were calculated as described in Materials and Methods from the plot of ln((α^4)^/(1 − α)) (insets of panels A and B). Δ*G_f_* and Δ*V_f_* correspond, respectively, to the *y*-intercept and the slope of the regressions presented in the insets (Table 1). As shown in Table 1, SUL stabilized the tetramers of WT-TTR and L55P by 2 and 8 kcal/mol, respectively, while IND promoted 6 and 11 kcal/mol stabilization (see ΔΔ*G_f_*).

Unfortunately, we do not have a precise definition as to which part of TTR is dismantled under high pressure. What we do know is that most of the secondary content of TTR (~45%) remains under pressure as revealed by FT-IR [51]. Also, we have evidences that the thyroxin channels are lost upon compression, since bis-ANS, which binds into these channels, are released upon compression.

As shown in Table 1, Δ*V_f_* values increased in the presence of both compounds for WT-TTR and L55P alone. Thus, more contacts (mainly electrostatic and van der Walls) must be broken in their presence in order to dissociate the tetramers into monomers. These findings will be explored in light of the crystallographic data presented next.

### 2.2. SUL, IND and LUM Inhibit the HHP-Induced Aggregation of WT-TTR and L55P

**Figure 3:**
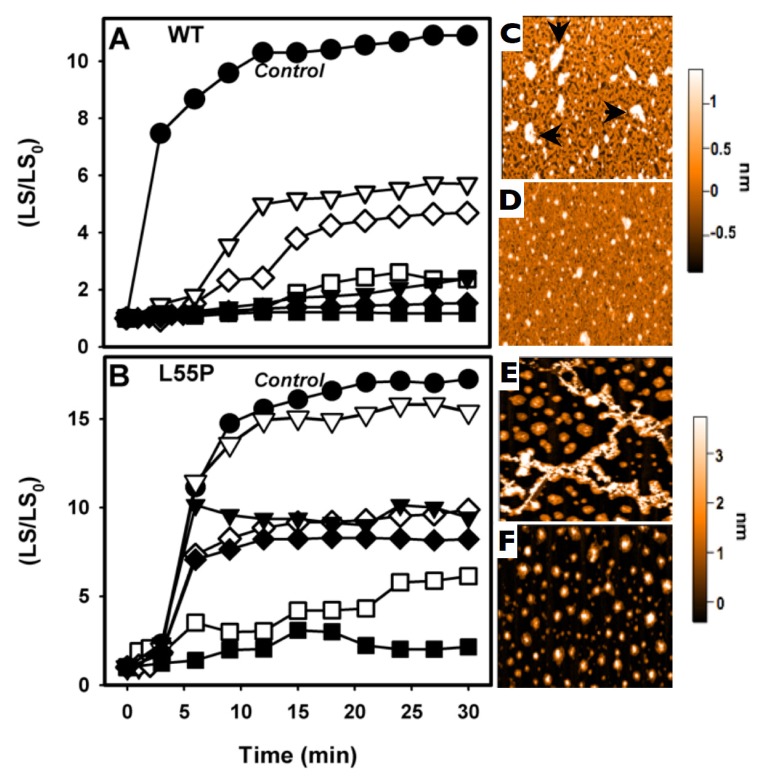
LUM, SUL and IND inhibit HHP-induced aggregation. WT-TTR (panel **A**; 3.5 μM) and L55P (panel **B**; 1 μM) were compressed for 1 h at 3 kbar at 1 °C, pH 5, in the absence or presence of LUM, IND or SUL. After this time, pressure was removed, and the temperature was increased to 37 °C, at which aggregation is triggered. Then, the light scattering (LS) was recorded and normalized to the initial LS value. Aggregation in the absence of any addition is shown as circles, while LUM is shown as squares, IND as diamonds and SUL as triangles. Filled symbols refer to a TTR:compound molar ratio of 1:10, while hollowed symbols represent the 1:2 TTR:compound molar ratio. LS was collected by exciting the samples at 320 nm and colleting the scattered light from 315 to 325 nm. Immediately after the end of the aggregation kinetics, an aliquot of each sample was mounted onto mica and analyzed by AFM. WT-TTR and L55P (panels **C** and **E**, respectively) aggregated in the absence of any addition, and WT-TTR and L55P (panels **D** and **F**) aggregated in the presence of 8 and 2 μM LUM (1:2 molar ratio).

We have shown that, after a cycle of compression–decompression at pH 5.5, TTR undergoes aggregation, forming amyloid fibrils [27,31]. This protocol is very useful for drug screening, because it is fast (aggregation is completed in less than 1 h) and can be performed at more amenable pH values (pH 5–5.5), avoiding exposition of the compounds under investigation to very low pH values. Thus, we evaluated whether the three compounds studied here would be able to inhibit the HHP-induced aggregation of TTR. To pursue this, WT-TTR (3.5 μM) or L55P (1 μM) were initially subjected to 3 kbar at 1 °C for 60 min (here called the dissociation-denaturation phase) in the absence or presence of LUM, IND or SUL (1:10 or 1:2 TTR:compound molar ratios). Under these conditions, tetramers were fully dissociated under pressure, exposing tryptophan to the aqueous environment (see Figure 2). After decompression, an altered, aggregation-prone tetramer (T_4_*) was formed, and as soon as the temperature was increased to 37 °C, aggregation was triggered and light scattering increased (Figure 3). This phase is called the aggregation phase, and Figure 3 shows examples of the aggregation of WT-TTR (panel A) and L55P (panel B). Aggregation of the proteins in the absence of any addition is shown in filled circles in each panel (control). As in the case of the WT-TTR, aggregation was completely blocked by the three compounds when they were used at a 1:10 ratio (filled symbols), and this was only partially inhibited at a lower TTR:compound ratio (1:2; hollowed symbols), although LUM (hollowed squares) inhibited WT-TTR aggregation almost completely, even at this low dosage. In the case of L55P (panel B) at a 1:10 molar ratio (filled symbols), aggregation was only partially inhibited (~45%) in the presence of SUL (filled triangles) and IND (filled diamonds) but was completely blocked by LUM (filled squares). With a lower L55P:compound regimen (1:2), SUL was completely ineffective (hollowed triangles) in inhibiting L55P aggregation, while IND (hollowed diamonds) and LUM (hollowed squares) inhibited only 36% and 65%, respectively.

To characterize the morphology of the aggregates formed after HHP treatment in the absence or in the presence LUM, AFM imaging was performed (Figure 3C–F). The aggregates composed of WT-TTR grown in the absence of any addition had a large, amorphous appearance measuring approximately 1 nm in height (arrows, Figure 3C), while the proteins that aggregated in the presence of LUM (1:2) presented a more homogeneous appearance, the aggregates were scarce (Figure 3D). Although the aggregates composed of WT-TTR that formed after HHP-treatment in the absence of any addition presented a non-fibrillar morphology, they bound thioflavin T and Congo red, while the species formed in the presence of LUM did not bind to these amyloid-specific probes (not shown).

In the case of L55P, the aggregates formed after pressure treatment in the absence of any addition presented the typical fibrillar appearance with heights of >3 nm (Figure 3E). Interestingly, as shown in panel F, the species formed in the presence of 2 μM LUM (1:2 molar ration) displayed a spherical, homogeneous appearance with different heights but were on average approximately 3 nm.

**Figure 4:**
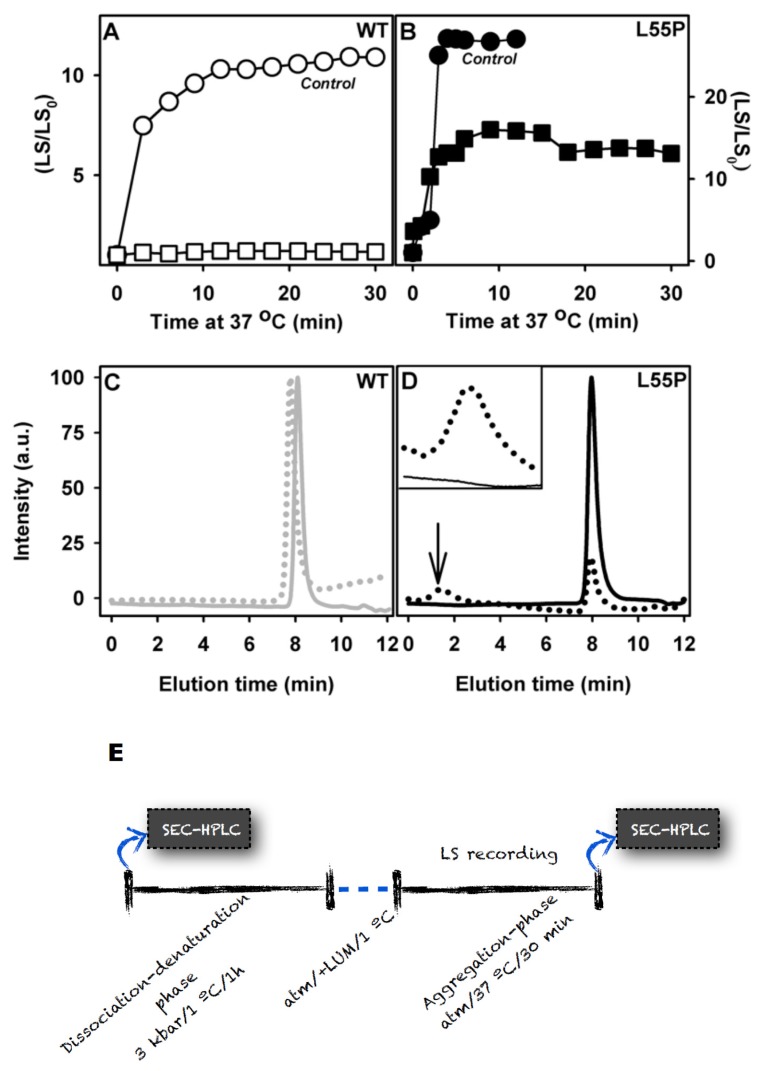
LUM completely inhibits the aggregation of T_4_*-WT-TTR but only partially inhibits the aggregation of T_4_*-L55P. Three-point-five micro molar per liter WT-TTR (panel **A**) or 1 μM L55P (panel **B**) were compressed at 3 kbar, 1 °C, pH 5, for 60 min in the absence of any addition. After this time, the pressure was released, the samples were kept on ice and LUM was added in a 1:10 TTR:LUM molar ratio. In the control samples, the same volume of buffer was added. Then, the temperature was raised to 37 °C to trigger aggregation, and the light scattering was recorded (panels **A** and **B**). Circles represent the aggregation of WT-TTR or L55P in the absence of any addition, while squares represent the aggregations after the addition of LUM. The oligomeric state of the species of WT-TTR (panel **C**) or L55P (panel **D**) formed after aggregation phases was evaluated by SEC-HPLC (dotted line). The continuous line shows the profiles of the samples before compression, where a homogenous population of tetramers was observed. For clarity, Panel E shows the experimental scheme adopted here.

The experiments reported thus far included the inhibitors in all phases, specifically the dissociation-denaturation phase (compression at 3 kbar/1 h at 1 °C) and the further aggregation phase (return to atmospheric pressure, 37 °C). Because these compounds are stronger tetramer stabilizers (Figure 2), combined with the impossibility of evaluating the effect of LUM on tetramer stability due to methodological limitations (explained in Section 1), this prompted us to speculate whether the observed inhibitory effect on the aggregation induced by HHP is due to the impossibility of populating the previously characterized species termed T_4_*, which is aggregation-prone, after pressure. Accordingly, we adopted an alternative protocol to evaluate the effects of LUM, which was the most effective aggregation inhibitor, on T_4_* aggregation. In this alternative protocol, the dissociation-denaturation phase was performed exactly as reported before, but the proteins were compressed in the absence of LUM, which allowed their full denaturation. Then, the pressure was decreased back to atmospheric levels, the high-pressure cuvette was opened on ice (to avoid aggregation), LUM (1:10) was added and the temperature was raised to 37 °C, triggering aggregation (see the scheme presented in Figure 4E). The results of these experiments (aggregation-phase) are presented in Figure 4A (WT-TTR) and B (L55P). As shown in the figure, the addition of LUM (1:10 molar ratio TTR:compound) completely inhibited the aggregation of T_4_*-WT-TTR but only partially inhibited the aggregation of T_4_*-L55P (52%). The precise explanation for this difference is not currently known, but it is possible that the conformation of T_4_*-L55P was more aggregation-prone than T_4_*-WT-TTR, a very plausible hypothesis given that native L55P is already more amyloidogenic that WT-TTR. Indeed, 1 μM of L55P gave rise to the same amount of aggregates as 3.5 μM of WT-TTR (see the LS values in panels A and B). It is also possible that T_4_*-L55P presented a lower affinity for LUM than T_4_*-WT-TTR. In either case, LUM would have acted as a weaker aggregation inhibitor for T_4_*-L55P than for T_4_*-WT-TTR.

In order to evaluate the oligomeric state of the species formed in the presence of LUM, aliquots were withdrawn and injected into a SEC-HPLC (Figure 4C,D). WT-TTR eluted as a tetramer after the aggregation phase in the presence of LUM (panel C, dotted line), confirming the complete inhibition of the aggregation of T_4_*-WT-TTR shown in panel A. In the case of L55P (panel D), the population of tetramers diminished to only 20% after aggregation in the presence of LUM with the concomitant appearance of a population eluting at 1.5 min, which was compatible with the size of large aggregates (dotted line and inset).

### 2.3. SUL, IND and LUM Inhibit Acid-Induced Aggregation of WT-TTR and L55P

**Figure 5:**
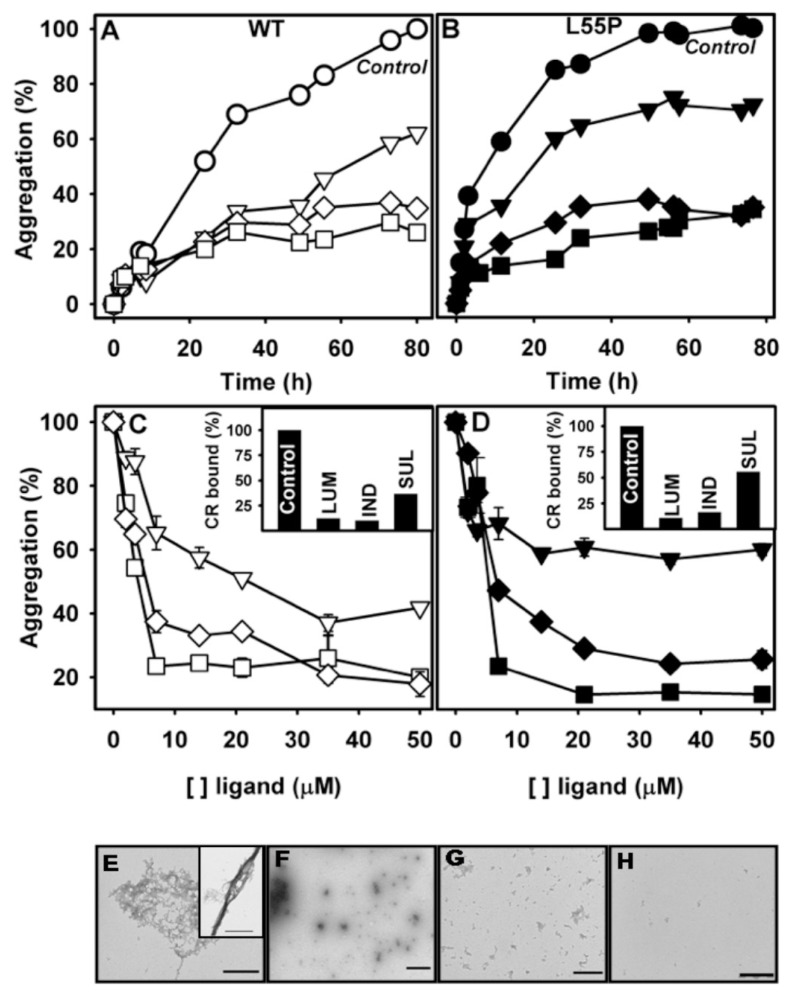
LUM, SUL and IND inhibit the acid-induced aggregation of TTR in a dose-dependent manner. WT-TTR (panel **A**, 3,5 μM) and L55P (panel **B**, 1 μM) were incubated in the absence (circles) or presence of LUM (squares), IND (diamonds) or SUL (triangles) at a molar ratio of 1:2 TTR:compound at pH 4.4 and 37 °C, and turbidity (400 nm) was evaluated over time. One hundred percent aggregation was assigned to the turbidity of the samples aggregated in the absence of any compound after 80 h under aggregating conditions. Panels **C** (WT-TTR) and **D** (L55P) show the extent of aggregation as measured by turbidity in the presence of increasing concentrations of the compounds (dose-dependent curves) LUM (squares), IND (diamonds) and SUL (triangles). The insets of panels **C** and **D** show the percent of Congo red binding to the samples aggregated in the absence or presence of each compound after 72 h under aggregating conditions. The morphology of WT-TTR aggregates after 72 h under acidic conditions was analyzed by TEM; Control (panel **E**) and in the presence of 8 μM SUL (panel **F**), 8 μM IND (panel **G**) or 8 μM LUM (panel **H**). Note the presence of amyloid fibrils in the control sample and their absence when aggregation was performed in the presence of the three compounds.

As shown before by several groups, the incubation of TTR under acidic conditions (pH ~4.0) leads to its aggregation over time, even in static solutions [23,26,51]. In the previous section, we showed that LUM, IND and SUL were effective in inhibiting the HHP-induced aggregation of TTR at pH 5.5, and of these LUM was the most effective compound. Thus, we performed aggregation kinetics with WT-TTR and L55P (Figure 5A,B, respectively) at pH 4.4 for 72 h in the absence (circles) or presence of all three compounds. In contrast to other amyloidogenic proteins, the aggregation process of TTR is devoid of a nucleation phase [51], as tetramer dissociation is the rate-limiting step in TTR fibrillation. The presence of the three compounds did not change this typical profile, which is important, because for the proteins in which nucleation phase is present, the small soluble oligomers that accumulate in the nucleation phase have been shown to be toxic species to cells in culture in several cases [52–55]. The three compounds inhibited the aggregation of both proteins in a 1:2 molar ratio, and LUM (squares) and IND (diamonds) were almost equally effective. Again, SUL (triangles) was the poorest inhibitor, particularly for L55P, whose aggregation was inhibited by SUL by only 30%.

Next, we performed dose-dependent curves for each compound and protein, and the data are presented in panels C and D. For this purpose, the proteins were incubated at pH 4.4 and 37 °C for 72 h in the absence or in the presence of increasing concentrations of the compounds; after this incubation time, the turbidity at 400 nm was measured. From these experiments, it is possible to see that LUM inhibited ~80% and ~90% of WT-TTR and L55P aggregation, respectively, when present in a 2:1 molar ratio (LUM:protein). IND also inhibited WT and L55P-TTR aggregation with high efficacy but at higher molar ratios (5:1 and 10:1, respectively). SUL was again the least efficient inhibitor among the three, inhibiting 60% of WT-TTR aggregation (10:1 molar ratio) and only 40% of L55P aggregation, even at high compound:protein ratios.

From the curves presented in Figure 5C,D, it was possible to calculate the IC_50_ for LUM and IND, which were equal to 5.2 and 7.40 μM, respectively, for WT-TTR and 8.4 and 11.7 μM for L55P. Due to the poor efficacy of SUL, it was not possible to calculate its IC_50_ index.

In other to confirm the amyloid architecture of the aggregates formed in the absence or presence of these compounds, the binding of Congo red (CR) was evaluated (insets of panels C and D) [56,57]. While the samples that aggregated in the absence of any additionally bound CR confirmed their amyloid nature, the samples that aggregated in the presence of the three compounds displayed much lower or no CR binding. While LUM inhibited 87% and 89% of the amyloid formation in the case of WT-TTR and L55P, respectively, IND inhibited 90% and 84%, while SUL inhibited 63% and 44%.

To confirm the reduction in fibril formation, TEM images of WT-TTR aggregated in the absence or presence of the three compounds were collected and are presented in panels E–H. In the control sample (WT-TTR alone; panel E and inset), fibrillar aggregates were abundant as expected, while the samples containing SUL (panel F), IND (panel G) and LUM (panel H) were completely devoid of any mature fibril, although some amorphous heterogeneous aggregates could be observed but in low amounts. Similar images were obtained with L55P (not shown).

As mentioned before, TTR aggregation presupposes the dissociation of tetramers into partially unfolded monomers that are aggregation-prone [23,24]. Thus, we wondered whether the effects of these compounds would be dependent exclusively on the presence of tetramers in solution and consequently on the occupancy of the HBS by the compounds, or whether they could have an additional mechanism of action, regardless of the tetramer population in solution.

**Figure 6:**
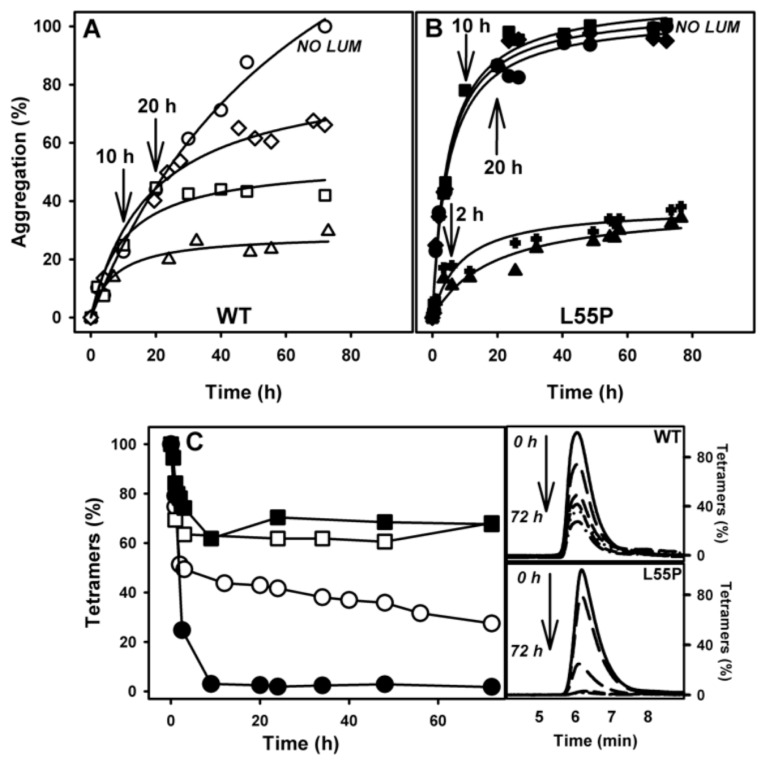
The inhibitory activity of LUM depends on the presence of tetramers in solution. 1 μM WT-TTR (panel **A**) and L55P (panel **B**) were incubated at pH 4.4 and 37 °C in the absence (circles) or presence of 8 μM LUM (triangles). In parallel, solutions with the same protein concentrations were left to aggregate in the absence of LUM. After 2 h (crosses), 10 h (squares) or 20 h (diamonds), 8 μM LUM was added. Aggregation was monitored by turbidity and was expressed as the percent of aggregation, setting as 100% the aggregation of the solutions in the absence of LUM at 72 h. Panel C shows the percentage of tetramers present during the aggregation kinetics experiment for WT-TTR (hollowed symbols) and L55P (filled symbols) in the absence (circles) or presence of 8 μM LUM (squares). It is possible to see that peak decreases corresponding to the elution of tetramers as aggregation proceeds in the chromatograms (samples aggregated in the absence of LUM) shown on the right.

To address this question, WT-TTR and L55P were incubated under aggregating conditions in the absence of LUM, which was added to the aggregation reaction at various times (2, 10 or 20 h) (Figure 6A,B). As a control, a solution containing 8 μM LUM added at the start of the kinetics experiment was left to aggregate and again showed the stronger inhibitory activity of LUM (triangles); another control solution was left to aggregate in the absence of any addition (circles).

Note that the addition of 8 μM LUM (1:2 LUM:WT-TTR) after 10 h (square) or 20 h (diamond) of the onset of WT-TTR aggregation (panel A) led to a progressive decrease in the activity of LUM as an inhibitor (compare with the curve represented with triangles). In the case of L55P aggregation (panel B), LUM (1:2) was able to inhibit aggregation only when added 2 h after the onset of the aggregation reaction (crosses). The addition of LUM after 10 h (squares) or 20 h (diamonds) of the onset of aggregation was ineffective, and these samples aggregated similarly to a sample incubated in the absence LUM (compare with the curve in circles). The lack of inhibitory activity when LUM was added later could have been due to the disappearance of the tetramers from the solution, because tetramers are the species that bind these compounds.

With the aim of monitoring the disappearance of the tetramers during the aggregation kinetics of WT-TTR and L55P, these proteins were left to aggregate, and several aliquots were withdrawn at various times and injected into an SEC to evaluate the amount of tetramers still present in solution. The disappearance kinetics of the WT-TTR tetramers (panel C, hollowed circles) and L55P (panel C, filled circles) during aggregation differed significantly. While ~50% of the WT-TTR tetramers persisted even after 10 h under aggregation conditions (pH 4.4), at this time point the L55P tetramers had already disappeared and were completely converted into aggregates. Even after 72 h under aggregating conditions 30% of WT-TTR tetramers remained in solution (panel C, hollowed circles), but there was a tendency for their complete disappearance at longer time points. Panel C also shows the percentage of tetramers in solution when aggregation was performed in the presence of LUM from the beginning of the aggregation reaction (squares). LUM stabilized the tetramers, impeding their dissociation into monomers and further conversion into amyloid aggregates. The chromatograms from which the amounts of tetramers presented in panel C were calculated are shown in the inset of panel C. The results of SEC-HPLC of WT and L55P samples performed over time from 0 to 72 h of aggregation are shown in the upper and lower panels.

Taken together, these data suggest that tetramer stabilization is the mechanism through which LUM exerts its inhibitory effects. Once tetramers have dissociated into monomers, LUM can no longer inhibit the aggregation of TTR. This was confirmed by using an engineered monomer of TTR, M-TTR [25], for which aggregation was not inhibited by LUM, even at high concentrations (not shown).

### 2.4. The Species Formed When Aggregation Is Performed in the Presence of LUM, IND or SUL Are Not Toxic to Cells in Culture

**Figure 7:**
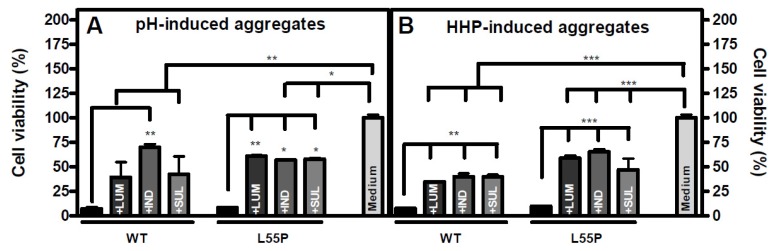
The species formed in the aggregation pathway in the presence of LUM, IND or SUL are innocuous to N2a cells. Aggregates composed of WT-TTR and L55P were produced either under acidic conditions (pH 4.4, 37 °C, 72 h; panel **A**) or by HHP-treatment (3 kbar at pH 5 for 1 h; panel **B**), either in the absence or in the presence of LUM, SUL and IND in a molar ratio of 1:2. Then, N2a cells were exposed for 24 h to 4 μM of each aggregate, and cell viability was evaluated by MTT reduction assays. The percentages of viable cells were calculated, setting the cells treated only with culture medium as 100% viable. No toxic effect was observed when cells were treated with each of the three compounds at up to 35 μM. Statistical analysis was performed in three independent experiments using one-way ANOVA with Tukey’s test, and *** *p* < 0.001; ** *p* < 0.01 and * *p* < 0.05.

As shown, the aggregation of TTR induced by HHP or acidic pH in the presence of LUM, IND or SUL generated species with a non-fibrillar appearance as visualized by AFM (Figure 3C–F) and EM (Figure 5E–H) imaging. Aiming to address whether these species would be toxic to cells in culture, especially in light of the current hypothesis that soluble small oligomers of the fibril formation pathway are the toxic species in amyloidoses [52–55,58–60] we evaluated their toxicity to human neuroblastoma (N2a) cells using MTT assays (Figure 7).

Aggregates of WT-TTR and L55P were produced by HHP treatment or after 72 h at pH 4.4 as previously described, either in the absence or presence of LUM, IND or SUL (1:2 TTR:compound, molar ratio). Then, 4 μM of these aggregates were incubated with N2a cells for 24 h, and MTT assays were performed. As shown in Figure 7, while the acid- or HHP-induced aggregates (Figure 7A,B, respectively) composed of WT-TTR or L55P were very toxic to these cells (~90% of the cells were not viable), the species formed in the presence of the three compounds were much less toxic to this cell line (only 30%–60% of the cells were not viable). Other TTR stabilizers were shown to have similar effects in preventing TTR aggregate cytotoxicity [54,61,62]. This lower toxicity was probably related to the fact that these inhibitors blocked fibril formation by stabilizing the tetramers, which seemed to be the major population when aggregation was performed in their presence (Figures 3 and 6C). The tetramers coexisted with a small population of amorphous aggregates that, when present, seemed to be innocuous as well. It is possible that these amorphous aggregates do not share the toxic properties of the soluble, on pathway aggregates and could be considered as off pathway species. Further studies are necessary to clarify this point. It is important to stress that the experiments shown in Figure 7 were performed with the total fraction of the aggregated material. In the case of the experiments performed in the absence of any addition, this suspension might include the remaining tetramers and monomers, all sort of soluble oligomers and mature fibrils. We believe that the toxicity observed with these samples arises mainly from the soluble oligomers still present.

### 2.5. Crystal Structures of WT-TTR in Complex with LUM, SUL, and IND

In order to gain insight into how the three compounds interact with TTR, we solved the crystal structure of WT-TTR in the absence and presence of the compounds studied here for the first time.

**Figure 8:**
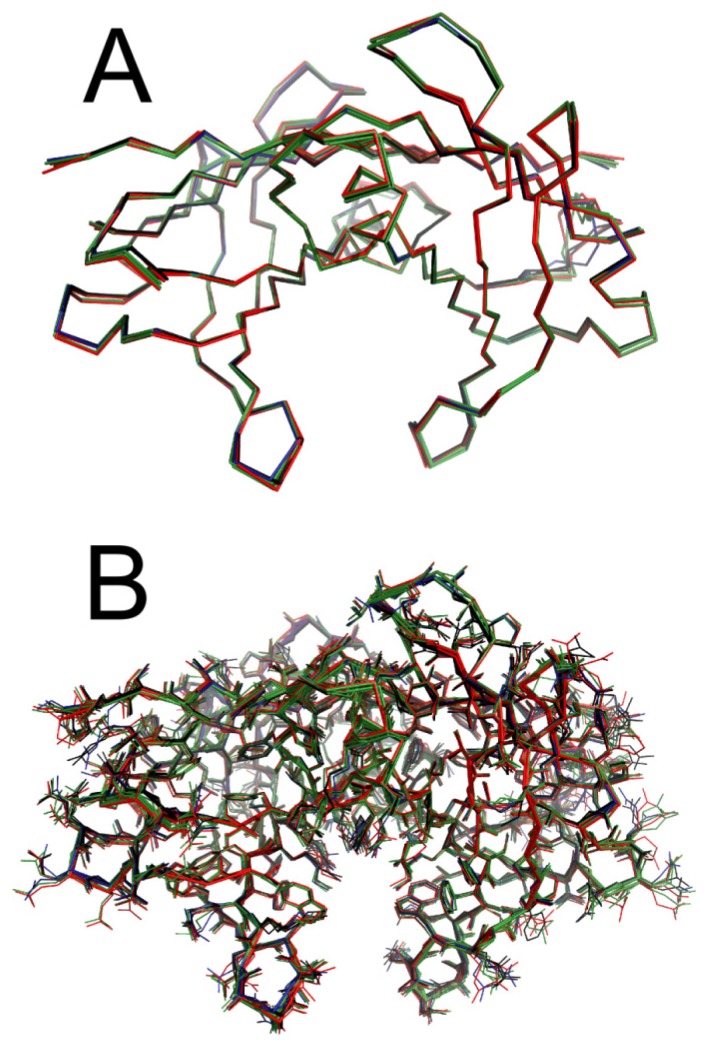
Overall view of the TTR crystal structures. The TTR crystal structure was solved in the apo form (red) and in the presence of LUM (green), IND (blue) and SUL (black), and the structures of the dimer from the asymmetric unit were superposed for purposes of comparison. (**A**) Backbone; (**B**) Backbone and side chains.

The structures of WT-TTR in the apo and holo forms were solved at high resolution, and all belonged to space group P2_1_2_1_2 with a dimer in the asymmetric unit (Table S2). All structures solved here were isomorphous, with 0.5 Å or less in overall RMSD of Cα distances between the superposed TTR structures using the apo WT-TTR as reference (Figure 8A); this indicated that there were no significant overall conformational changes upon ligand binding. Moreover, with the exception of a few amino acids in the HBS involved in ligand recognition, residue side chains were mostly in similar conformations for TTR in the absence or presence of any of the three compounds (Figure 8B).

**Figure 9:**
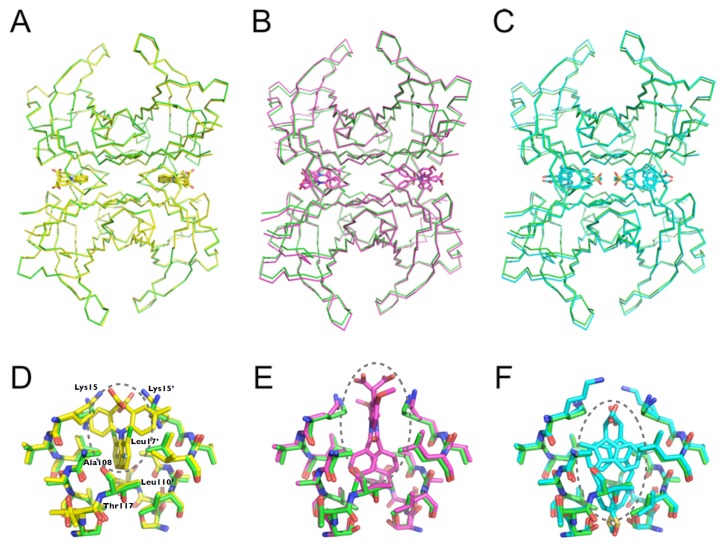
Ligand binding to TTR. The crystal structure of the TTR tetramer was generated by the application of symmetry operations and superposed for evaluating the conformation and ligand binding. (**A**–**C**) Backbone representations of the superposition of apo TTR (green) and TTR in complex with (**A**) LUM (yellow), (**B**) IND (magenta) and (**C**) SUL (cyan). (**D**–**F)** Detailed views of one of the two HBS of the apo TTR (green) and in complex with (**D**) LUM (yellow), (**E**) IND (magenta) and (**F**) SUL (cyan).

**Figure 10:**
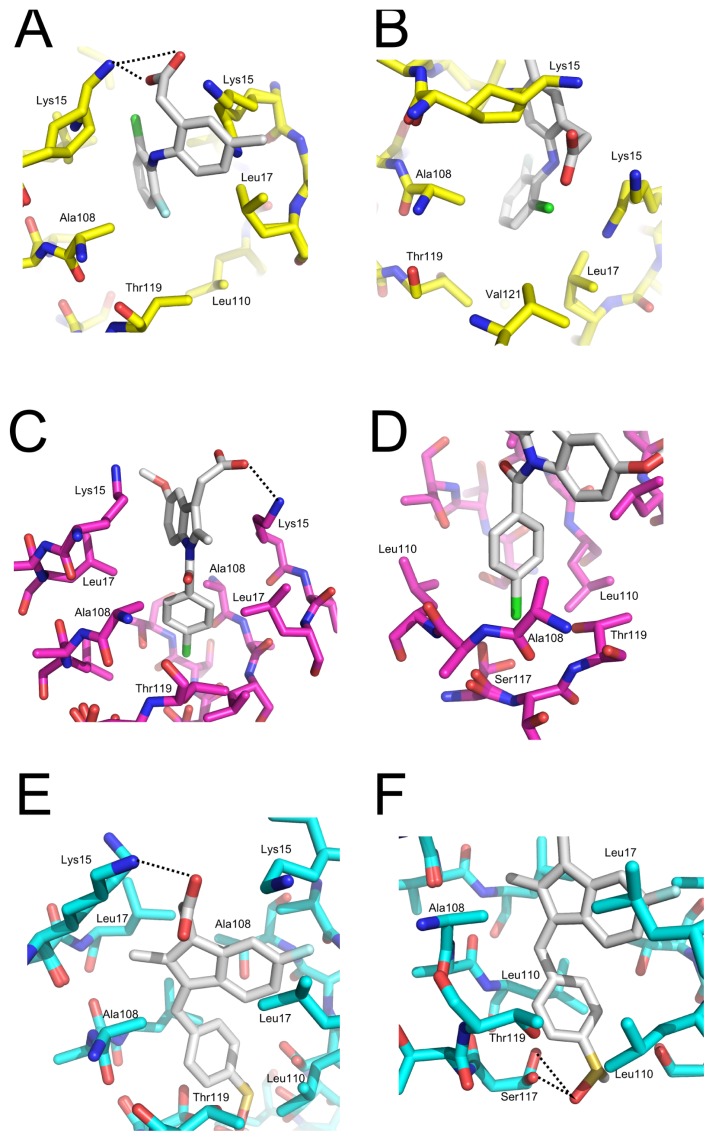
Details of the interactions and positioning of LUM, IND and SUL into binding pockets. Backbone representations of HBS interactions with LUM (**A**,**B**), IND (**C**,**D**) and SUL (**E**,**F**). The residues involved in more important interactions are enumerated. Distances between side chains and ligand interactions are depicted with blue dotted lines, and distances are measured in angstroms.

All ligands were present in both HBS located at the dimer-dimer interface (Figure 9A–C). The ligands were modeled at 0.5 occupancy in both HBS due to the two-fold symmetry operator at this channel axis. SUL, IND and LUM all shared the common feature of being anchored by electrostatic contact with Lys15 through their carboxylic moiety (Figures 9 and 10). Ligand binding to the HBS seemed to be identical, as judged from the superposition of the monomers (not shown). We will discuss further details of their interaction with TTR.

Details of WT-TTR:LUM interaction—LUM is an analogue of diclofenac; its chlorine is substituted by a fluorine atom, and a methyl group replaces a hydrogen atom in the phenylacetic moiety. Diclofenac has long been investigated as a model for the design of analogues targeting TTR [42,43]. Both diclofenac and diclofenac analogues bind to TTR in the so-called “reverse mode”, *i.e.*, with the phenylacetic moiety occupying the inner binding cavity [42]. We have recently shown that diclofenac binds to TTR in at least two alternate modes, with the dihalogen-substituted ring rotated at about 90 degrees taking the phenylacetic moiety as a positional reference [43]. In contrast to diclofenac, LUM bound to TTR in the “forward mode”, *i.e.*, with the phenylacetic moiety facing the outer binding cavity, contacting simultaneously both Lys15 and Lys15’ (Figure 9D). The LUM fluorine atom was inserted in inner halogen binding pocket 3 (HBP3), a hydrophobic cavity formed between the side chains of Thr119, Ala108, Leu110 and Leu17’ (Figure 10A), while the chlorine atom of LUM was located at a pocket adjacent to HBP2 lined by Leu17 (which rotates upon LUM binding to TTR), Val121, Ala108’ and Thr119’ (Figure 10B).

LUM binding to TTR led to the formation of a stable hydrophobic cavity between Leu17 and Lys15 that was filled by a water molecule in spite of the expected low probability of occupancy of non-polar cores with water. The energetic cost of such an interaction might have been compensated for by the highly favorable interaction of LUM with TTR, which demands an inductive effect (that is, the effect of charge transmission through a chain of atoms in a molecule) in this region, allowing the anchoring of halogen atoms from LUM at this site. Moreover, hydration of this cavity did not seem to be costly, as suggested by the existence of a water molecule in a partially occupied region in the apo form of TTR. Collectively, the intrinsic dynamics of Lys15 and Leu17 and the inductive characteristic of this cavity, as suggested by the recent identification of this site [43], reinforced the possible use of these features in the optimization of lead compounds targeting TTR.

Details of the WT-TTR:IND interaction—the carboxyl group of IND was anchored by an electrostatic interaction with Lys15 (Figure 10C). Lys15’ generated a positive electrostatic environment that supported a close proximity to the 5-metoxyl group of IND. The methylene from Thr-119 formed a hydrophobic pocket (HBP3) in conjunction with Leu110 into which the chlorine group of IND was inserted (Figure 10D). The methoxyl group was stacked in the HBP1 between Leu17 and Val121, while the indolic methyl group was stacked in the HBP1’ in the opposing monomer comprising the HBS.

Considering the observed proximity of the opposing Lys15’ to the methoxyl group, substitution of the latter by an acidic group could favor interaction with TTR due to the introduction of an additional contact between them. Moreover, this interaction would be enhanced by improvements in the van der Waals contact between Leu17 and Val121 through functionalization at the methylene group.

Details of the WT-TTR:SUL interaction—SUL bound more deeply in the HBS than IND, about 4 Å toward the tetramer center, and the molecule (in particular its indenyl ring) was rotated perpendicularly about 90° when compared to the indol group from IND (Figure 9C). SUL interaction with TTR was mediated by van der Waals contacts and a few polar bonds. The carboxyl group from SUL contacted with Lys15 through an electrostatic interaction approximately 3 Å apart (Figure 10E). The SUL keto group interacted directly with the hydroxyl group from Ser117, mediated by a hydrogen bond 3.2 Å apart, and with the hydroxyl group from Ser115 through a water molecule, while the methylene from the methylsulfonyl group performed a van der Waals interaction with Leu110 (Figure 10F). The SUL rotational orientation inside the channel seemed to be directed by the binding of the fluorine and methylene groups to the central halogen binding pockets (HBP2) and the symmetry-related HBP2, comprised by side chains of Lys15, Leu17, Ala108 and Leu110. Moreover, both Thr119 and Ser117 side chains were in close proximity to the phenyl moiety of sulindac, increasing the interaction potential at this region.

The three NSAI compounds investigated here are very dissimilar to each other. Although they belong to the same therapeutic class, these three compounds do not constitute an exact homologous series. The most prominent characteristic of the interactions of these ligands with TTR is the presence of their ionizable carboxyl group, which is able to bind directly to the epsilon-amino group of Lys15; this amino acid is located at the entrance of the HBS and serves as both a polar anchoring lock for polarizable functional groups as well as a hydrophobic patch through its hydrocarbonate moiety, which constitutes the HBP.

Both LUM and IND make the most extensive contact with TTR and are involved in electrostatic interactions with Lys15 and stack into two HBP. SUL is inserted deep inside the channel, stacking its methylsulfonyl group between the two central Ser117, and is anchored by electrostatic contact with Lys15 and insertion into the HBP2. Taking into account the functional data obtained from the binding, stabilization and aggregation assays, it is suggestive that either more contacts with the HBP and binding involving less insertion into the channel might result in better TTR affinity and privileged effectiveness in preventing dissociation and aggregation. However, the present data also support the notion that TTR is still a particularly challenging case for rational drug design, because ligand binding and orientation inside the channel are not highly predictive based on previous data using homologous series; this was observed for the two distinctive orientations—forward and reverse—between diclofenac and lumiracoxib and the rotational orientation inside the channel as supported by crystallographic data with diclofenac [43] and the two analogs SUL and IND.

The effectiveness of these NSAI compounds in stabilizing TTR against amyloid aggregation described here motivates further investigation to their potential use as lead compounds against FAP.

Besides, the concentrations used here (micro molar per litre range), which were effective in inhibiting TTR aggregation, are below those used in clinics.

These data provide novel perspectives in the design of molecules targeting the HBP of TTR, and can assist in the establishment of a larger database comprising the crystallographic and functional data from prospective studies of ligand:TTR interactions aiming to assist in our understanding of the structure-activity relationship targeting TTR amyloidosis.

## 3. Experimental Section

### 3.1. Chemicals

All reagents were of analytical grade. Sulindac and indomethacin were kindly provided by Dr. Ana Luiza Palhares de Miranda (Faculdade de Farmácia, UFRJ, Brazil). Lumiracoxib was kindly provided by Dr. Tailane S. Moreira (Faculdade de Farmácia, UFRJ, Brazil).

All compounds tested as possible inhibitors were weighed and diluted in DMSO in order to reach the final concentration. The concentrations were confirmed by spectroscopy using the extinction coefficient of each compound. In all experiments using the compounds, the same volume of DMSO alone was added to the samples as a “blank control” to control for the effect of DMSO alone.

### 3.2. Protein Purification

WT-TTR and L55P were expressed and purified as described previously [5]. The TTR concentration was determined using an extinction coefficient of 7.76 × 10^4^ M^−1^ cm^−1^ at 280 nm.

### 3.3. Congo Red (CR) Binding Assay

As an independent measurement of aggregation and to confirm the amyloid nature of the aggregates formed, CR assays were performed. A known volume of the aggregated solution was mixed with CR solution in a 1:10 protein:CR molar ratio. The extent of amyloid formation was measured by the amount of CR bound to the species in solution in molar per litre (characterized by a red-shifted absorbance) and quantified by the equation: Equation 1:

(1) $$(\text{CR bound}/\text{L}\;\text{of amyloid suspension})={\text{A}}_{540\;\text{nm}}/25295-{\text{A}}_{477\;\text{nm}}/46306$$

### 3.4. Atomic Force Microscopy

Samples were diluted to a concentration of 1 μM in phosphate buffer, and a volume of 50 μL was allowed to adhere onto freshly cleaved mica for 10 min. Excess material was removed by blotting. The samples were washed 5 times with 200 μL Mili Q water and air dried overnight. AFM was performed in air in tapping-mode using an Asylum MFP-3D BIO AFM (Asylum Research, Santa Barbara, CA, USA) and Olympus rectangular silicon cantilevers with a resonance frequency of 70 kHz and nominal spring constant of 2 N/m. Images were obtained by scanning the samples at a rate of 0.5–1.0 Hz, and 512 × 512 pixels were collected in each image. Samples were exhaustively examined to confirm their homogeneity.

### 3.5. Aggregation Measurements

WT-TTR and L55P aggregation were induced by two different protocols as follows:

1. Acid-induced aggregation: 3.5 μM of WT-TTR and L55P tetramers were left to aggregate in 200 mM sodium acetate and 100 mM KCl at pH 4.4 and 37 °C in staticsolutions. At specific time points (legend of each figure) or after 72 h, turbidity at 400 nm was measured to evaluate the extent of aggregation. The compounds were added at the beginning of the aggregation reaction or at intermediate time points as stated in the legend of each figure. The efficacy of each compound was calculated as 100% of the turbidity of the sample aggregated in the absence of any addition.
2. HHP-induced aggregation: WT-TTR at 3.5 μM and L55P at 1 μM were pressurized at 3 kbar for 1 h in MES 100 mM and KCl 100 mM at pH 5 and 1 °C (dissociation-denaturation phase). After this time, the pressure was released, and the temperature was quickly increased to 37 °C, at which time aggregation was triggered (aggregation-phase). Then, light scattering (LS) was measured over time to evaluate the extent of aggregation. Each LS measurement was normalized to the LS value before compression (LS/LS0). In several experiments, LUM, IND and SUL were added at the beginning of the experiment. In another protocol, LUM was added after the dissociation-denaturation phase, keeping the high-pressure cuvette on ice to avoid aggregation. Then, the temperature was increased to 37 °C to trigger aggregation, now in the presence of LUM.

An ISS spectrofluorometer (Champaign, IL, USA) was used for LS measurements, the samples were excited at 320 nm and light was collected at 90° through the monochromator from 315 to 325 nm.

### 3.6. Electron Microscopy

Samples were vortexed, and 5 μL of the suspension was absorbed onto 200 mesh carbon-coated copper grids for 5 min and blotted to remove excess material. Negative staining was performed by adding 2% uranyl acetate for 3 min and air drying. The grids were examined with a Jeol 1200 electron microscope (Jeol Ltd., Tokyo, Japan) operating at 60 kV.

### 3.7. Inhibitor Binding Measured by ANS Displacement

WT-TTR and L55P (1 μM) were incubated with 10 μM 1,8-anilinonaphthalene-8-sulfonic acid (ANS) in 25 mM Tris HCl and 100 mM KCl at pH 7 for 10 min. To measure ANS fluorescence, samples were excited at 370, and the emissions were collected from 400 to 600 nm in an ISS spectrofluorometer (Champaign, IL, USA). With the addition of each ligand, samples were allowed to equilibrate for 10 min before collecting the ANS spectrum. To calculate the percent of ANS displaced by the compounds, the following equation was used:

(2) $$D=100-[(F_{0}-F)/(F_{0}-F_{\text{b}})]\times 100$$

where *D* is the percent of ANS displaced; *F*_0_ is the ANS fluorescence intensity when it is free in solution; *F* is the ANS fluorescence intensity in a determined ligand concentration and *F*_b_ is the initial fluorescence intensity of ANS when it fully bound to TTR.

### 3.8. Size Exclusion Chromatography (SEC)

Quantification of WT-TTR and L55P tetramers during acid-induced aggregation or after HHP treatment was performed by SEC. 50 μL of a previously centrifuged sample (10,000 rpm for 10 min to remove insoluble material) were injected into a GPC 300 column (Amersham Biosciences, Piscataway, NJ, USA) linked to an HPLC system (Shimadzu, Japan). Protein elution was followed by fluorescence and absorbance. The percent of tetramers present at each time point or for each condition was inferred by calculating the area of the tetramer peak with consideration to the area at time zero (soluble tetramers) set as 100% of tetramers.

### 3.9. Neuroblastoma Cell Culture and Viability Assay (N2a)

N2a cells were cultured in Dulbecco’s modified Eagle’s medium supplemented with 10% fetal bovine serum and 2% antibiotic (gentamicin) in a 5% CO_2_ atmosphere for 3 days and then transferred to a 96-well plate and allowed to adhere for 24 h (5,000 cells/well). After adhesion, 20 μL of medium from each well were removed and replaced by 20 μL of the aggregated sample (acid- or HHP-induced aggregates, total fraction). In these experiments, the aggregates were generated by incubating 20 μM of TTR in the absence or presence of 40 μM inhibitors. After dilution, the final concentration in each well was 4 and 8 μM (TTR:inhibitors), respectively. Cells were challenged for 24 h, and final cellular viability was measured by an MTT assay as previously described [54]. The cytotoxicity of all compounds at concentrations of 3.5 to 35 μM was achieved by the same protocol, and no impact on cell viability was observed.

### 3.10. Stability Studies under HHP

The high-pressure cell equipped with optical windows was previously described [46] and was purchased from ISS (Champaign, IL, USA). Fluorescence spectra were recorded on an ISS K2 spectrofluorometer (ISS Inc., Champaign, IL, USA). The pressure was increased in steps of 200 bar. At each step, the sample was allowed to equilibrate for 15 min prior to taking measurements. Tryptophan emission spectra were obtained by setting the excitation at 280 nm and collecting the emission in the 300–400 nm range. The mean energy of the fluorescence emission at pressure p was evaluated by the center of spectral mass (ν_p_) as follows:

(3) $$<\nu_{\text{p}}>=\Sigma \nu_{\text{i}}F_{\text{i}}/\Sigma F_{\text{i}}$$

where *F*_i_ is the fluorescence emitted at a wavelength ν_i_. The degree of dissociation (α) is related to ν_p_ by the equation:

(4) $$\alpha =(\nu_{\text{i}}-\nu_{0})/(\nu_{\text{d}}-\nu_{0})$$

where ν_i_ is the center of spectral mass of the tryptophan emission at each pressure value, and ν_0_ and ν_d_ are, respectively, the center of spectral mass of the folded and of the unfolded proteins.

### 3.11. Thermodynamic Parameters

The free-energy change of folding was extracted from the HHP experiments according to the equation:

(5) $$\Delta G_{f}=-\text{R}T\;\text{ln}K_{f}$$

where *K_f_* is calculated from the slope of the curve of ln(α^4^/(1 − α)), in which α is the degree of denaturation. The standard volume changes for folding (Δ*V_f_*) for the WT-TTR and L55P were calculated according to:

(6) $$\text{ln}(\alpha^{4}/(1-\alpha ))=\Delta \;V_{f}/\text{R}T\;p+\text{ln }K_{u}$$

where *R* is the gas constant, *p* is high pressure, *T* is the temperature at which the experiment was performed and K_u_ is the unfolding constant. The Δ*V_f_* is extracted from the slope of the curve, while *K_u_* is the intercept in the *y*-axis [47].

### 3.12. WT-TTR Crystallization, Data Diffraction and Structure Solution

TTR crystals in the apo form (no ligand) were obtained by the hanging drop vapor-diffusion method. Diffraction-quality crystals were obtained in about 5 days under two different crystallization conditions. TTR complexes with ligands were prepared by soaking apo WT-TTR crystals into mother liquor supplemented with saturating amounts of the compounds for at least 30 minutes before data collection under a nitrogen stream at 100 K as follows:

1. We grew crystals from 2 μL drops containing 5 mg/mL TTR, 11% peg 400, 50 mM Tris and 50 mM KCl, equilibrated at 18 °C against 22% peg 400, 100 mM Tris pH 7.4 and 100 mM KCl, and collected data with CuKα radiation (1.5418 Å) generated by a Rigaku UltraX 18 rotating anode operated at 50 kV and 90 mA, equipped with Osmic confocal Max-Flux optics and recorded on a MAR 345dtb image plate mounted over a mar dtb (MAR Research);
2. We grew crystals from 2 μL drops containing 5 mg/mL TTR, 14% *v*/*v* peg 400, 50 mM Hepes sodium pH 7.5 and 100 mM CaCl_2_, equilibrated at 20 °C against 28% *v*/*v* peg 400, 100 mM Hepes sodium pH 7.5 and 200 mM CaCl_2_ (directly from Hampton’s Crystal Screen screening kit, condition #14) and collected data with the D03-MX1 beamline from LNLS (National Synchrotron Light Laboratory, LNLS, Campinas, Brazil; [63], where the wavelength was set to 1.427 Å and the diffraction intensities were measured using a MARCCD detector mounted over a mar345dtb (MAR Research).

X-ray diffraction data were collected with 1° oscillation per image. The main objective in collecting more than one dataset from different crystals whenever possible under different growing conditions and with different data collection strategies was to ensure that the models converged on a representative structure and to eliminate the uncertainty and bias from a single data set [64]. These two crystallization conditions resulted in apo WT-TTR crystals with equivalent conformations. These data support the notion that WT-TTR displays a well-defined configurational state [65]. We used both crystallization solution conditions for further characterization of the TTR:ligand complexes to rule out eventual effects rising from chemical adjuvants and buffers over the crystal structures of TTR, both in the apo form and in complex with sulindac, indomethacin and lumiracoxib.

Indexing and processing was performed with Mosflm, and the integrated images were merged and scaled with Scala [66]. The structure of apo WT-TTR was solved by molecular replacement with MolRep [67] using an apo form of human WT-TTR as a search model input (PDB ID 3CFM). Structure refinement was conducted with Refmac [68] and Coot [69] which were also used for the automated and manual addition of water molecules. Ligands were manually added for the refined structures using structures and libraries generated in the ProDRG2 server [70] (http://davapc1.bioch.dundee.ac.uk/prodrg/).

Atomic locations are precise to better than 0.25 Å (Table S2). Structural validation of the model was performed with PROCKECK [71] and showed all main-chain dihedral angles in the allowed regions with all residues found in favorable Ramachandram regions. Due to the 2-fold symmetry of the binding sites, each ligand molecule was refined at 0.5 of occupancy and was partially superposed with its copy generated by the two-fold symmetry. Omit maps were generated with the Omit program [72] from the CCP4 suite [66,68]. All figures were generated with PyMOL.

## 4. Conclusions

Fortunately, life expectancy has increased in the last decade worldwide. However, with this increase in longevity, several diseases are becoming prevalent; among them are the amyloid diseases, with special emphasis on Alzheimer’s and Parkinson’s diseases. FAP is the most prevalent systemic amyloidosis and is caused by the deposition of TTR variants, mainly in peripheral nerves. Up until now, there has been no cure for these diseases. Therefore, the discovery of new compounds that inhibit their aggregation is urgent. TTR is the secondary carrier of the thyroid hormone in blood plasma, and over 99% of thyroxine binding channels in TTR remain unoccupied *in vivo*; therefore, they are suitable targets for drug development. Here, we studied the inhibitory activity of LUM, IND and SUL against TTR aggregation using WT-TTR and L55P, a very aggressive variant. The first two compounds were very effective in inhibiting the aggregation of both proteins in a dose-dependent manner. The species formed when aggregation was performed in their presence were shown to be much less toxic to neuroblastoma cells in culture. These compounds bind to the native tetrameric structure of both WT-TTR and L55P, stabilize them and prevent their further dissociation into monomers, which are the building blocks for amyloid formation. The HHP-induced aggregation protocol, which is very rapid, was successfully employed for the first time and shown to be very promising for the selection of potential candidates as TTR-aggregation inhibitors. The crystallographic data presented here highlighted the particular characteristics of ligand interaction with TTR at the HBS, such as halogen bonding between ligand and TTR, the importance of complimentary binding to Lys15, and fulfillment of the hydrophobic (halogen) binding pockets, as well as direct interactions with Ser117 and Thr119. We envision that highly complementary ligand binding to TTR could be achieved through the collective use of these binding features in the design of novel ligand scaffolds, ultimately resulting in the enhancement of both binding specificity and affinity.

## Supplementary Information



## Figures and Tables

**Table 1:** Thermodynamic parameters for the association-folding of WT-TTR and L55P in the absence or presence of SUL and IND. These data were extracted from Figure 2 and calculated according to Equations (5) and (6) in Material and Methods.

	WT			L55P		
		
	Apo	SUL	IND	Apo	SUL	IND
ΔCM (nm)	5.2	5.2	3.9	6.8	6.0	6.3
p_1/2_ (bar)	980	1240	2607	514	932	1824
Δ*G_f_* (kcal/mol)	−16.01 ± 0.3	−17.98 ± 0.25	−21.90 ± 0.35	−24.60 ± 0.6	−32.40 ± 0.75	−35.30 ± 0.8
ΔΔ*G_f_* (kcal/mol)	-	1.96 ± 0.2	5.89 ± 0.05	-	7.8 ± 0.15	10.7 ± 0.2
Δ*V_f_* (mL/mol)	175.5 ± 15.56	241.0 ± 19.8	264.40 ± 9.89	275.10 ± 14.85	326.0 ± 12.02	407.0 ± 12.73
