# Supplementary material for: Inhibition of Human Transthyretin Aggregation by Non-Steroidal Anti-Inflammatory Compounds: A Structural and Thermodynamic Analysis
Source: Int J Mol Sci. 2013 Mar 6;14(3):5284–311. doi: 10.3390/ijms14035284 (PMC3634512; doi:10.3390/ijms14035284)
Supplement: Supplementary file 1 [file ijms-14-05284-s001.docx]

Supplementary Information

**Table S1.** Apparent dissociation constants for NSAI compounds. *K_d_* values were calculated from the data in Figure S1.

| **NSAI compound** | **WT** | **L55P** |
| --- | --- | --- |
| LUM | 1.4 ± 0.2 µM | 1.7 ± 0.2 µM |
| IND | 3.5 ± 0.9 µM | 6.7 ± 0.9 µM |
| SUL | 5.1 ± 1.1 µM | 5.1 ± 1.1 µM |

**Figure S1.** Evaluation of the equilibrium of NSAI compounds binding to TTR. Ligand binding was measured as described previously [43] by the displacement of the fluorescent probe ANS. WT (panel **A**) or L55P (panel **B**) TTR at 1 μM was incubated and equilibrated with ANS at 2 μM, increasing concentrations of compounds (LUM, squares; IND, diamonds and SUL, triangles) were added to the samples and the change in ANS fluorescence was measured. Ligand binding resulted in ANS displacement from thyroxine channels with a concomitant decrease in fluorescence emission. The apparent *K_d_* was calculated by using a one-site saturation function as previously described [43].


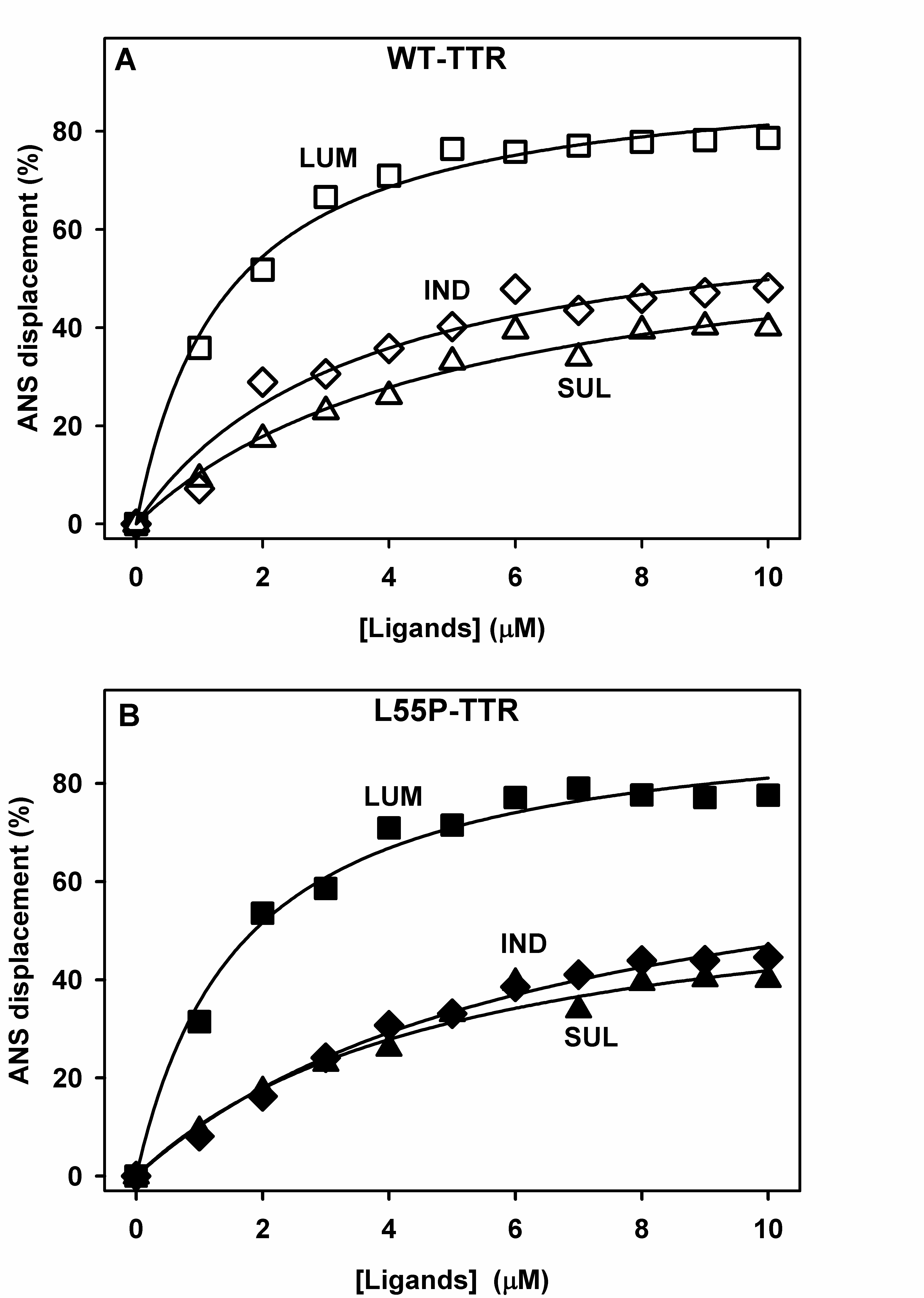


**Table S2.** Data collection and refinement statistics for the apo and complexed forms of the WT-TTR.

| **PDB entry** | **3W3B** | **4IKL** | **4IKJ** | **4IKK** | **4IKI** | **4IK7** | **4IIZ** | **4IK6** |
| --- | --- | --- | --- | --- | --- | --- | --- | --- |
| Ligand | apo | Sulindac | Sulindac | Sulindac | Indomethacin | Indomethacin | Lumiracoxib | Lumiracoxib |
| Radiation source | Synchrotron | CuKα | Synchrotron | Synchrotron | CuKα | Synchrotron | Synchrotron | Synchrotron |
| Space group | P2_1_2_1_2 | P2_1_2_1_2 | P2_1_2_1_2 | P2_1_2_1_2 | P2_1_2_1_2 | P2_1_2_1_2 | P2_1_2_1_2 | P2_1_2_1_2 |
| Resolution range, Å | 21.8–1.90  (1.95–1.90) | 42.8–1.90  (1.95–1.90) | 63.9–2.1  (2.16–2.10) | 19.8–2.1  (1.95–1.90) | 63.5–2.00  (2.05–2.00) | 21.8–2.10  (2.15–2.10) | 21.7–2.10  (2.15–2.10) | 21.7–2.00  (2.05–2.00) |
| Unit cell dimension, Å | 42.4, 83.5, 63.5 | 43.1, 85.5, 64.1 | 42.4, 83.5, 63.9 | 41.5, 83.1, 637 | 43.1, 86.1, 63.5 | 42.0, 83.6, 63.0 | 41.9, 83.0, 63.0 | 41.9, 83.2, 62.8 |
| Monomers/asymmetric unit | 2 | 2 | 2 | 2 | 2 | 2 | 5.6 | 2 |
| Multiplicity | 3.2 (3.1) | 6.7 (6.7) | 3.0 (2.9) | 3.0 (2.9) | 6.5 (6.4) | 3.4 (3.4) | 3.2 (3.2) | 3.3 (3.3) |
| Completeness, % | 97.0 (97.0) | 100 (100) | 95.9 (95.9) | 95.9 (95.9) | 99.4 (99.4) | 99.9 (99.9) | 99.8 (99.8) | 99.8 (99.8) |
| I/σ (I) | 19.9 (3.2) | 19.1 (3.4) | 12.0 (4.4) | 12.0 (4.4) | 20.4 (4.3) | 12.0 (3.0) | 20.6 (4.0) | 18.7 (3.1) |
| Rmerge, % | 5.7 (25.4) | 7.4 (54.0) | 7.7 (23.1) | 7.7 (23.1) | 7.1 (41.0) | 8.5 (37.7) | 5.6 (26.0) | 6.0 (32.8) |
| Structure refinement |  |  |  |  |  |  |  |  |
| No. of reflections used in refinement | 16,837 (1,163) | 18,338 (1,333) | 12,405 (938) | 16,572 (1,162) | 15,622 | 12,850 (901) | 12,654 | 14,611 (1,056) |
| No. of reflections used for R_free_ | 909 (71) | 990 (76) | 777 (55) | 1,043 (59) | 830 (67) | 667 (45) | 658 (44) | 766 (47) |
| R_work_/R_free_, % | 17.9 (25.6)/23.5 (34.4) | 20.4 (29.2)/24.4 (29.2) | 19.8 (22.2)/25.7 (22.3) | 19.9 (27.9)/24.9 (33.4) | 17.1 (31.9)/23.5 (31.9) | 20.2 (24.4)/24.4 (27.8) | 17.2 (16.0)/23.9 (20.7) | 19.2 (23.2)/23.8 (28.5) |
| B factor, Å |  |  |  |  |  |  |  |  |
| Wilson | 23.5 | 23.7 | 24.6 | 23.7 | 24.1 | 29.2 | 31.4 | 30.1 |
| Overall | 28.8 | 23.1 | 24.1 | 25.5 | 24.9 | 27.7 | 31.2 | 32.1 |
| Protein | - | | | | | | | |
| Ligand |  |  |  |  |  |  |  |  |
| Water |  |  |  |  |  |  |  |  |
| Ramachandran outliers | 0/231 | 1/231 | 1/231 | 1/231 | 0/231 | 0/231 | 0/231 | 0/231 |
| Rmsd bond length, Å | 0.019 | 0.010 | 0.011 | 0.011 | 0.019 | 0.008 | 0.017 | 0.009 |
| Rmsd bond angles, ° | 2.03 | 1.36 | 1.50 | 1.49 | 2.13 | 1.37 | 2.13 | 1.46 |

Number in parentheses indicate the highest resolution shell. R_free_ calculated for ≥5% randomly chosen reflections that were excluded from the refinements.
